# Characterization of the first *Pseudomonas grimontii* bacteriophage, PMBT3

**DOI:** 10.1007/s00705-021-05173-0

**Published:** 2021-08-04

**Authors:** Sabrina Sprotte, Erik Brinks, Natalia Wagner, Andrew M. Kropinski, Horst Neve, Charles M. A. P. Franz

**Affiliations:** 1 Department of Microbiology and Biotechnology, Max Rubner-Institut, Federal Research Institute of Nutrition and Food, Hermann-Weigmann-Str. 1, 24103 Kiel, Germany; 2 Departments of Food Science and Pathobiology, University of Guelph, 50 Stone Road E, Guelph, Ontario N1G 2W1 Canada

## Abstract

The complete genome sequence of the virulent bacteriophage PMBT3, isolated on the proteolytic *Pseudomonas grimontii* strain MBTL2-21, showed no significant similarity to other known phage genome sequences, making this phage the first reported to infect a strain of *P. grimontii*. Electron microscopy revealed PMBT3 to be a member of the family *Siphoviridae*, with notably long and flexible whiskers. The linear, double-stranded genome of 87,196 bp has a mol% G+C content of 60.4 and contains 116 predicted protein-encoding genes. A putative tellurite resistance (*terB*) gene, originally reported to occur in the genome of a bacterium, was detected in the genome of phage PMBT3.

**Supplementary Information:**

The online version contains supplementary material available at 10.1007/s00705-021-05173-0.

## Introduction

*Pseudomonas grimontii* was originally isolated from French natural mineral water in 2002 [1]. The bacterium also appears to be associated with soil and has been reported to cause turnip bacterial rot disease in Japan [2]. As an environmental microorganism capable of biofilm formation, *P. grimontii* may become problematic in the food industry. Currently, there are more than 300 genome sequences of *Pseudomonas* phages available in the National Center for Biotechnology Information (NCBI) and European Nucleotide Archive (ENA) databases, with no phages so far having been isolated specifically from *P. grimontii*. Moreover, numerous studies have demonstrated the successful application of *Pseudomonas* phages or their endolysins against plant [3–7] and human [8–13] pathogens. However, only a few studies have investigated phages against strains of *Pseudomonas* species that cause food spoilage [14–16]. In this study, we present an analysis of the genome sequence of the novel virulent phage PMBT3, which was isolated from sewage at a municipal wastewater treatment plant located close to Kiel in Germany. The phage was detected in a double-layer agar assay using *P. grimontii* strain MBTL2-21 as the host bacterium. Since large volumes of drinking water are routinely used in the dairy industry for cleaning purposes, strains of this species may be introduced post-pasteurization into dairy environments [17]. In addition, phage PMBT3 showed lysis of *Pseudomonas* strains that are associated with milk spoilage. Therefore, phage PMBT3 could have the potential to make dairy foods safer and improve their shelf-life. To our knowledge, this is the first report of a bacteriophage infecting *P. grimontii*.

## Materials and methods

The proteolytic activity of *P. grimontii* MBTL2-21 was tested with 50 µl of overnight-grown culture spotted on skim milk agar prepared as described by Kazanas [18] and incubated at room temperature (20 °C to 22 °C) overnight. Afterwards, the strain was used in a phage screening assay, and for this, a sewage sample obtained from the local municipal wastewater treatment plant located in Bülk, close to the city of Kiel, was filtered through a 0.45-µm-pore-size membrane filter (Whatman^®^ FP30, Schleicher & Schuell, Dassel, Germany). Five milliliters of the filtrate was combined with 0.1 ml of *P. grimontii* MBTL2-21 (overnight culture), 1 mM CaCl_2_, and 10 ml of Caso broth (Carl Roth, Karlsruhe, Germany). After incubation for 18 h, the mixture was again filtered (0.45 µm), and 0.1 ml was spotted onto a lawn consisting of 0.3 ml of *P. grimontii* strain MBTL2-21 overnight culture and 1 mM CaCl_2_ in 3 ml of molten Caso soft agar (Caso broth with 0.7% [w/w] agar) poured onto Caso agar (Carl Roth, Karlsruhe, Germany). After incubation for 18 h at room temperature, the phage-produced lysis spot was scraped off the plate, and the phage was subjected to three successive rounds of single-plaque purification as described previously [19]. For propagation of phage PMBT3, 10 plates from plaque assays with confluent lysis were selected. The plates were each washed with 3 ml of modified SM buffer (0.58% NaCl, 0.25% MgSO_4_ × 7H_2_O, 0.24% Tris-HCl [pH 7.4]) [20] for 2 h at 200 rpm. After this, the lysates were pooled and filtered through 0.45-µm-pore-size membrane filters. This lysate was further concentrated and purified using cesium chloride (CsCl) density gradient ultracentrifugation as described by Sambrook and Russell [20].

Transmission electron microscopic analysis of a high-titer lysate of the phage was performed using a Tecnai 10 transmission electron microscope (FEI Thermo Fisher, Eindhoven, The Netherlands) as described elsewhere [21].

The host range of phage PMBT3 was evaluated using different *Pseudomonas* target species (Online Resource 1) in a modified overlay assay performed in duplicate. Briefly, 0.3 ml of bacterial culture grown to an OD_620_ of 0.3 was combined with 0.1 ml of phage lysate, 0.25% (v/v) glycine, 10 mM CaCl_2_, and 10 mM MgCl_2_. After adsorption for 10 min at room temperature, 2.5 ml of Caso soft agar (0.35%) was added, and the mixture was poured onto Plate Count Agar (VWR, Darmstadt, Germany) supplemented with 0.25% glycine, 10 mM CaCl_2_, and 10 mM MgCl_2_. Finally, 10 µl of tenfold serially diluted phage lysates (prepared with phage dilution buffer consisting of ¼-strength Ringer's solution [Merck, Darmstadt, Germany] and 10% (v/v) Caso broth) was spotted onto the bacterial lawn, and the plates were incubated for 18 h, after which they were examined for phage-produced lysis zones or single plaques. Plates were usually incubated at room temperature (20 °C to 22 °C), except for those containing *Pseudomonas koreensis* (30 °C), *Pseudomonas rhodesiae* (30 °C), or *Pseudomonas aeruginosa* (37 °C), which were also used in the host range screening. The efficiency of plating (EOP) values for phage PMBT3 were calculated relative to the titer of this phage on the original host strain MBTL2-21 (3×10^9^ plaque-forming units [pfu] per ml).

Phage DNA was isolated from 2 ml of high-titer phage lysate using a Phage DNA Isolation Kit (Norgen Biotek Corp., Thorold, Canada) according to the manufacturer's protocol. DNA was quantified and adjusted using a Qubit 3.0 fluorometer (Invitrogen, Germany). For DNA library preparation and genome sequencing, a Nextera XT Library Preparation Kit and a MiSeq Reagent Kit V2 were used according to the manufacturer's instructions on a MiSeq high-throughput sequencer (Illumina, Munich, Germany). The raw sequence data were evaluated and assembled *de novo* using Shovill 1.0.9 [22]. Open reading frames (ORFs) were predicted automatically using Rapid Annotation using Subsystem Technology (RAST) [23] and then analyzed manually for their putative functions using BLASTp [24] and SMART [25]. Their locations and predicted functions are shown in Online Resource 2. Putative host-dependent promoters (TTGACA[N15-19]TATAAT) in intergenic regions were identified using Kodon (Applied Maths, Austin, TX, USA), allowing a maximum of a 2-bp mismatch. Putative rho-independent terminators were identified using ARNold [26] at http://rssf.i2bc.paris-saclay.fr/toolbox/arnold/, with those occurring in intergenic regions and possessing a free energy in the stem-loop region of < -10 kcal mol^-1^ being retained. Their locations and sequences are shown in Online Resource 3. The amino acid sequences of the putative terminase large subunit (TerL) and tellurite resistance proteins (TerB) from PMBT3 were compared to the most closely related proteins using BLASTp. An amino acid sequence alignment of these protein sequences with selected related proteins encoded in the genomes of bacteria and other phages (GenBank accession numbers [GBANs] are listed in Supplementary Tables S5 and S6 in Online Resource 4) was performed in Geneious version 11.0.2 using the Geneious aligner [27]. A comparison of the phage PMBT3 genome to other phage genomes published in the databases (NCBI and ENA) was performed at the nucleotide level with megablast on the BLASTn platform [24]. Furthermore, the Virus Classification and Tree Building Online Resource (VICTOR) [28] (freely available at https://victor.dsmz.de) was used for whole-amino-acid-sequence-based phylogeny and classification. For this purpose, the phage PMBT3 proteome was compared to that of its closest relative, *Pseudomonas* phage Lana [29], and 30 *Pseudomonas* phages in the databases, with a focus on *P. fluorescens* phages, including the recently described *P. fluorescens* phage PMBT14 [30] (GBANs and their characteristics are listed in Supplementary Table S7 in Online Resource 4). All pairwise amino acid sequence comparisons were conducted using the Genome-BLAST Distance Phylogeny (GBDP) method with optimal settings as described by Meier-Kolthoff et al. [28]. More precisely, taxon boundaries were estimated with the clustering thresholds for species (i.e., 0.118980), genus (i.e., 0.749680), and family (i.e., 0.985225) [28]. Automatically generated phylogenetic trees were rooted at the midpoint [31] and visualized using FigTree [32].

## Results

**Fig. 1:**
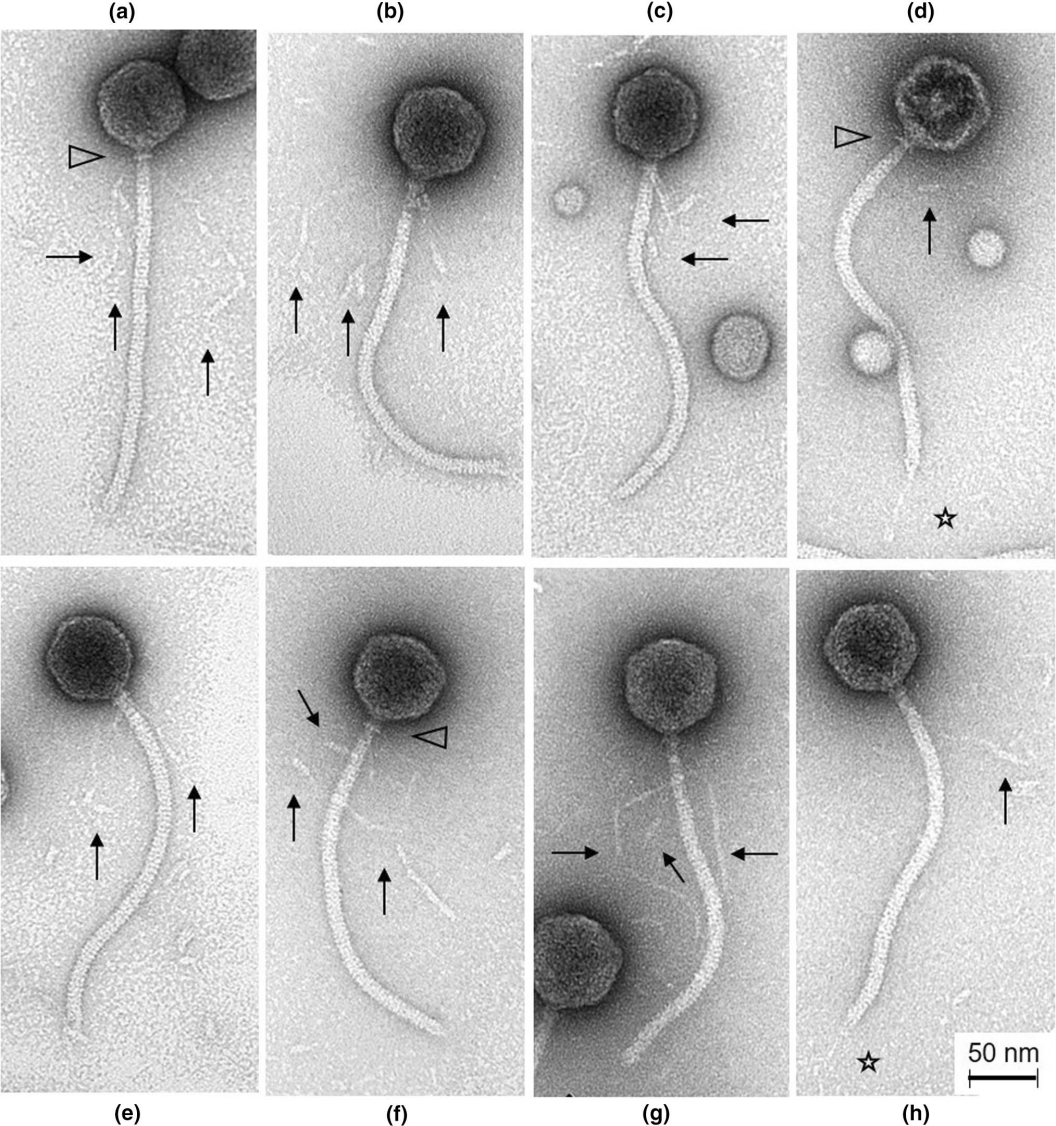
Transmission electron micrographs of *Siphoviridae* phage PMBT3 of *P. grimontii* strain MBTL2-21. The arrows indicate the notably thin and highly flexible long whiskers attached to a distinct collar (neck passage) structure (see triangles). The asterisks indicate a thin tail fiber structure visible on the phage particles on the right (d and h).

*P. grimontii* MBTL2-21 is capable of hydrolyzing casein on skim milk agar (proteolysis assay; data not shown). Therefore, this strain was selected as a presumable milk-spoilage strain for isolation of lytic phages. The isolated phage PMBT3 produced small (0.5 mm in diameter), turbid plaques on *P. grimontii* strain MBTL2-21 lawns grown on Caso soft agar at room temperature. Electron microscopy and measurement of dimensions of 22 phage particles revealed a morphotype with an isometric head of 69 ± 2.3 nm in diameter and a 268 ± 5.1-nm-long, non-contractile and flexible tail (Fig. 1). These characteristics indicate that phage PMBT3 belongs to the family *Siphoviridae.* A thin neck passage (nps) or collar structure (width 15.5 ± 0.9 nm) is indicated by triangles in Fig. 1a, d, and f. Notably, the phage showed up to three extraordinarily long and highly flexible whiskers (154 ± 13.3 nm) adhering at the nps structures that were found at various random positions in the vicinity of the tails. Occasionally these appendages were also detected in a bent loop formation (Fig. 1c). The distal ends of these whisker structures were also shaped with cylindrical extensions of variable length (Fig. 1a, b, c, f, h). A thin central tail fiber (length: 57 ± 8.6 nm) at the conical tail tip is indicated by asterisks in Fig. 1d and h.

In the host range assay, phage PMBT3 showed not only lytic activity against *P. grimontii* strain MBTL2-21 but also against several *P. fluorescens* strains (4 out of 7 strains tested) isolated from food samples and against *P. koreensis* R05-1 (1 strain tested), *P. lactis* strains G-8961 and G-8962 (2 out of 3 strains tested), *P. protegens* G-number 9102 (1 out of 3 strains tested), and *P. rhodesiae* B03-5 (1 strain tested) (Online Resource 1). The phage produced distinct (pinpoint) plaques on its original host strain MBTL2-21 and one tested *P. fluorescens* strain 17-L-08580-2-1, while it only formed cleared lysis zones on other *Pseudomonas* spp. strains. However, EOP values were significantly reduced for four *P. fluorescens* strains (10^-2^ to 10^-6^), one *P. rhodesiae* strain (10^-4^), one *P. koreensis* strain (10^-3^), two *P. lactis* strains (10^-4^), and one *P.* *protegens* strain (10^-6^).

**Fig. 2:**
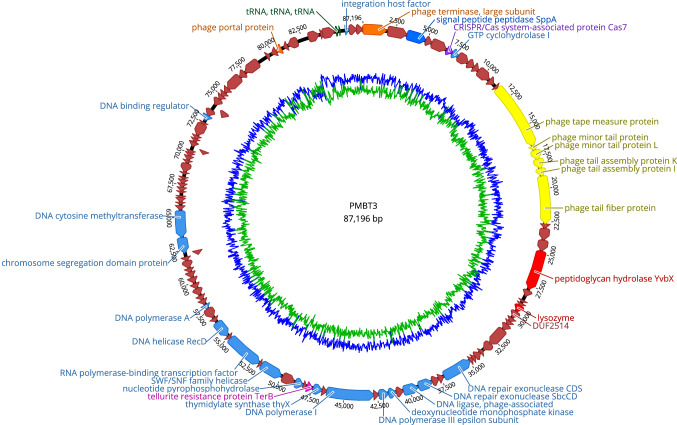
Genome map of PMBT3 (87,196 bp) with structural and functional annotations and GC content displayed in the inner circle. The genome starts with the ORF coding for the putative terminase large subunit (ORF1) shown at the top of the map. The putative tellurite resistance gene (*terB*) between the predicted replication and transcription modules is indicated by a pink arrow. The three tRNA genes in the postulated transcription and replication module are also shown. An automated annotation was obtained from RAST, and all ORFs were also annotated manually using BLASTp and SMART. The genome was subdivided into functional modules as indicated by different colors (for details, see legend). The map was generated using Geneious 11.0.2.

Genome sequencing of phage PMBT3 produced 526,502 reads. A total of 518,833 paired-end reads were assembled *de novo* using Shovill 1.0.9 [22] into a single contig with a length of 87,196 bp. The DNA sequence had a mol% G+C content of 60.4. Automated annotation of the contig was performed using RAST [23], and this was followed by manual curation, resulting in 116 predicted open reading frames (ORF1-116) that contained both a start and stop codon, as well as a ribosome binding site (Online Resource 2). The ORFs were arranged in functional modules for DNA packaging, tail morphogenesis, DNA replication and transcription, and host lysis (Fig. 2). Amongst the total predicted open reading frames, only 31 ORFs (i.e., 27%) encoded proteins with a putative function, with only eight of these being assigned to phage-related proteins. The remaining 85 ORFs (i.e., 73%) were unclassified with no assigned category (i.e., encoding hypothetical proteins). The absolute numbers of start codons included 99 ATG, 11 GTG, and 6 TTG codons, respectively. Genomic analysis with PHACTS [33] revealed no lysogeny-related genes (e.g., genes encoding integrase, repressor, or antirepressor proteins), thus confirming the lytic nature of phage PMBT3. Using tRNAscan-SE version 2.0 [34], three tRNA genes for proline, glutamine, and methionine, respectively, were identified, and these were located between ORFs coding for DNA transcription and packaging proteins. Directly upstream of the thymidylate synthase gene (*thyX*) in the postulated replication module of PMBT3, a gene encoding a putative tellurite resistance protein (*terB*) was detected by BLASTp (Fig. 2) and verified using HHpred [35]. The encoded 150-amino-acid-long TerB protein showed the closest similarity to TerB proteins of bacteria, i.e., *Pseudomonas* sp. HMWF031 (GBAN PTU03211; coverage, 99%; identity, 60.4%) and *Pseudomonas* sp. B1(2018) (GBAN WP_116583222; coverage, 99%; identity, 61.7%), and less similarity to TerB of the first described member of the genus *Lanavirus*, i.e., the *Pseudomonas* phage Lana [29] (GBAN YP_009820378.1; coverage, 99%; identity, 50.3%). An amino acid sequence alignment with selected TerB proteins deduced from bacterial and phage genomes (i.e., e-values from 1e-53 to 3e-43) showed a distribution of homologous amino acids over the entire length of the protein, as demonstrated by the consensus sequence and identical amino acids in the individual sequences shown in Supplementary Fig. S1 in Online Resource 5. Structural analysis of the terminase large subunit protein using BLASTp [24] revealed a conserved domain belonging to the terminase GpA superfamily (pfam05876), which has also been detected in *cos*-site *Escherichia coli* phage lambda and *Salmonella* prophage Gifsy-2 [36, 37].

**Fig. 3:**
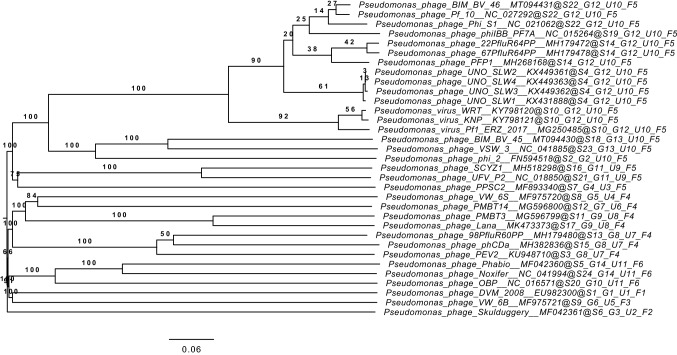
Phylogenomic analysis of phage PMBT3 with *Pseudomonas* phages from the NCBI database (GBANs are listed in Online Resource 4) at the amino acid level using VICTOR. The tree is based on the recommended formula D6 and shows GBDP pseudo-bootstrap support values from 100 replications above branches. The branch lengths of the resulting VICTOR tree are scaled in terms of the respective distance formula used.

A search of the databases using megablast [24] indicated that the phage PMBT3 genome showed only low similarity at the nucleic acid level (i.e., coverage, 35%; identity, 81.7%) to *Pseudomonas* phage Lana [29] (GBAN NC_048166 [38]), which has a similar genome size of 88,342 bp. The resulting amino-acid-based phylogenetic GBDP tree generated with VICTOR [28] for the phages PMBT3, Lana, PMBT14, and 30 other *Pseudomonas* phages is shown in Fig. 3. The tree was reconstructed with the formula D6 and yielded an average support of 71%. It shows a branch distance of about 0.23 between the closest relative phages PMBT3 and Lana. In addition, a comparison of the predicted terminase large subunit proteins from the most closely related bacteria (or prophages) and phages in the NCBI database with that of PMBT3 showed moderate sequence similarity (i.e., 81% identity) to the corresponding protein of phage Lana, but only low similarity (i.e., a maximum of 58% identity) to the corresponding proteins of bacteria and of other phages (Supplementary Fig. S2 in Online Resource 5).

## Discussion

*P. grimontii* strain MBTL2-21 might be a proteolytic strain, as it hydrolyzed casein when grown on skim milk agar. This characteristic is of relevance to the dairy industry, as it may cause milk spoilage. Other *Pseudomonas* spp., such as *P. fluorescens*, *P. gessardii*, *P. fragi*, *P. proteolytica*, *P. lundensis*, and *P. lactis* are known to be major milk spoilage microorganisms at low temperatures in raw milk bulk tanks in dairies (or in the refrigerator at home), based on their ability to produce proteolytic and/or lipolytic enzymes [39, 40]. *P. grimontii* may be introduced into the dairy environment via the washing water used in large volumes in dairies. Therefore, (heat-treated) milk may not play a significant role in the dissemination of these strains. The use of phages, which can eliminate such “problematic” *Pseudomonas* spp. strains during milk storage may thus prevent spoilage or product defects that might occur during subsequent milk processing. Phage PMBT3 is a potential candidate for this purpose, as the results of the host range assay showed that, in addition to the *P. grimontii* strain MBTL2-21, it also lysed a strain of *P. fluorescens*. Furthermore, it showed lysis zones when tested on strains of *P. koreensis*, *P. lactis*, and *P. rhodesiae*, the last of which belongs to the *P. fluorescens* group [41]. The reason why no single plaques were visible could be that phage PMBT3 has not been adapted to other strains. Alternatively, it was not able to infect the strains and the lysis zones were caused by lysis from without or by other toxic effects. Even though phage titers could not be determined with the various *Pseudomonas* strains (except for one *P. fluorescens* strain) due to the lack of single plaque formation, different efficiencies of plating when compared to the host strain could be identified.

Electron microscopy showed a unique feature of this phage, as it contained extraordinary long and highly flexible whiskers with so far unknown functions. Regarding whisker-encoding ORFs, we failed to identify any ORF coding for the two long whiskers in the phage PMBT3 genome. Furthermore, we could not assign any of the six hypothetical structural ORFs (i.e., ORFs 8-13) between the predicted DNA packaging and tail morphogenesis module to the function of a capsid protein. The head of PMBT3 thus seems to be constructed from completely different capsid proteins to those described previously for other phages. However, for phage tail morphogenesis, we found six ORFs located between ORF13 and ORF20, which encoded a tail tape measure protein (ORF14), a minor tail protein (ORF15 and ORF16), a tail assembly protein (ORF17 and ORF18), and a tail fiber protein (ORF19) (Fig. 2 and Online Resource 2), respectively. It has been suggested that tRNAs are randomly gained from their hosts and then lost either neutrally or according to a set of different selection mechanisms [42]. The fact that phage PMBT3 and its closest relative phage, Lana, harbor different numbers of tRNAs could be an explanation for their different target hosts (i.e., *P. grimontii* for PMBT3 and *Pseudomonas* sp., which, however, could also include *P. grimontii* for Lana). The putative tellurite resistance protein TerB (ORF50)-encoding gene in the genome of PMBT3 might have originated from a bacterial genome, which is supported by the fact that ORFs for similar gene products have been found in *Pseudomonas* spp. genomes (Supplementary Fig. S1 in Online Resource 5). Potassium tellurite (K_2_TeO_3_) was used as antimicrobial agent before modern antibiotics were introduced [43]. Two other phages that infect *Salmonella*, i.e., FSL SP-076 and FSL SP-058, have previously also been described to harbor a single TerB gene [44]. Furthermore, a TerB-like gene was also found in the genome of *Pseudomonas* phage Lana [29] (GBAN YP_009820378.1). All of these proteins, however, showed relatively low similarity to TerB of phage PMBT3, which shows more similarity to bacterial TerB gene products (Supplementary Fig. S1 in Online Resource 5). Since the *terB* gene detected in PMBT3 is not accompanied by other Tel^R^ genes, which are necessary for activity [45], and since neither a promoter (i.e., -35 and -10 region) nor a rho-independent terminator could be identified upstream and downstream of the gene, respectively (Online Resource 3), this putative TerB gene product is suspected to have no tellurite-resistance-mediating activity.

The genome sequence of PMBT3 exhibited low similarity (i.e., 35% coverage and 83% identity with the megablast algorithm or 57% coverage and 78% identity with the discontiguous megablast algorithm [29]) to only one phage in the NCBI database, i.e. *Pseudomonas* sp. phage Lana, and clustered with this nearest neighbor in an amino-acid-based phylogenetic tree. The branching value exceeded the distance of 0.12 that has been recommended as the cutoff value for phage species [28], indicating that phage PMBT3 represents a novel taxon, separate from the *Pseudomonas* sp. phage Lana taxon. In fact, new taxons have recently been proposed and approved by the International Committee on Taxonomy of Viruses (ICTV) for both phages, with phage PMBT3 as the sole member of the new genus *Maxrubnervirus* [46] and phage Lana as the type member of the new genus *Lanavirus* [29, 38] in the family *Siphoviridae*. Finally, a comparative analysis of the predicted terminase large subunit gene also confirmed the hypothesis that phage PMBT3 represents a new species (Supplementary Fig. S2 in Online Resource 5). The catalytically active ATPase in the terminase large subunit is a housekeeping gene product that is well suited for phylogenetic comparison between phages, since it appears to be relatively conserved in dsDNA phages [36]. It is the main component of the terminase holoenzyme and is required for the highly specific process of phage DNA packaging. In conclusion, phage PMBT3 is (so far) unique regarding its genome sequence as well as its morphology. To our knowledge, this is the first reported phage infecting a *P. grimontii* strain.

## Nucleotide sequence accession number

The complete genome sequence of phage PMBT3 generated in this project was deposited in the GenBank database (National Center for Biotechnology Information [NCBI]) under the accession number MG596799.1.

## Supplementary Information

Below is the link to the electronic supplementary material.

Supplementary file1 (PDF 54 kb)

Supplementary file2 (PDF 125 kb)

Supplementary file3 (PDF 111 kb)

Supplementary file4 (PDF 30 kb)

Supplementary file5 (PDF 725 kb)
