# Supplementary material for: Characterization of the first Pseudomonas grimontii bacteriophage, PMBT3
Source: Arch Virol. 2021 Aug 4;166(10):2887–94. doi: 10.1007/s00705-021-05173-0 (PMC8421299; doi:10.1007/s00705-021-05173-0)
Supplement: Supplementary file 1 — Supplementary file1 (PDF 54 kb) [file 705_2021_5173_MOESM1_ESM.pdf]

Supplementary Table S1. List of *Pseudomonas* spp. strains applied for host range analysis of PMBT3.

***Pseudomonas* species**	**Source**	**Phage titers (pfu/ml)**	**EOP values**
P. grimontii MBTL2-21 (host strain)	n.d.	$3x10^{9}$ [(a)]	1
P. fluorescens 17-L-08580-2-1	food	$4x10^{7}$ [(a)]	ca. $1x10^{-2}$
P. fluorescens 17-L-08880-2-1	food	ca. 10^{4} [(b)]	ca. $3x10^{-6}$
P. fluorescens 17-L-08582-1-1	food	ca. 10^{8} [(b)]	ca. $3x10^{-2}$
P. fluorescens 17-L-08580-1-1	food	ca. 10^{8} [(b)]	ca. $3x10^{-2}$
P. fluorescens 17-L-08128-3-1	food	[(c)]	[(d)]
P. fluorescens L1-61	n.d.	[(c)]	[(d)]
P. fluorescens MG-number 548	raw milk	[(c)]	[(d)]
P. rhodesiae B03-5	meat	ca. 10^{6} [(b)]	ca. $3x10^{-4}$
P. trivalis K05-9	n.d.	[(c)]	[(d)]
P. koreensis R05-1	n.d.	ca. 10^{7} [(b)]	ca. $3x10^{-3}$
P. cedrina B03-3	n.d.	[(c)]	[(d)]
P. brenneri K04-10	n.d.	[(c)]	[(d)]
P. gessardii R01-2	n.d.	[(c)]	[(d)]
P. gessardii G-number 9072	raw milk	[(c)]	[(d)]
P. gessardii MG-number 1376	raw milk	[(c)]	[(d)]
P. lactis G-number 8961	raw milk	ca. 10^{6} [(b)]	ca. $3x10^{-4}$
P. lactis G-number 8962	raw milk	ca. 10^{6} [(b)]	ca. $3x10^{-4}$
P. lactis DSM 29167, G-number 8927	raw milk	[(c)]	[(d)]
P. lundensis K05-3	n.d.	[(c)]	[(d)]
P. lundensis G-number 9036	raw milk	[(c)]	[(d)]
P. lundensis G-number 9108	raw milk	[(c)]	[(d)]
P. luteola L2-181	vegetables	[(c)]	[(d)]
P. meridiana MG-number 758	raw milk	[(c)]	[(d)]
P. meridiana MG-number 592	raw milk	[(c)]	[(d)]
P. protegens G-number 9102	raw milk	ca. 10^{4} [(b)]	ca. $3x10^{-6}$
P. protegens MG-number 967	raw milk	[(c)]	[(d)]
P. protegens MG-number 541	raw milk	[(c)]	[(d)]
P. proteolytica DSM 15321, G-number 8126	raw milk	[(c)]	[(d)]
P. proteolytica G-number 9043	raw milk	[(c)]	[(d)]
P. proteolytica MG-number 1412, G-number 9371	raw milk	[(c)]	[(d)]
P. aeruginosa L2-85	carrot	[(c)]	[(d)]
P. aeruginosa L2-108	raw milk	[(c)]	[(d)]
P. aeruginosa L2-109	raw milk	[(c)]	[(d)]
P. aeruginosa DSM 1117	n.d.	[(c)]	[(d)]
P. oryzihabitans L2-182	vegetables	[(c)]	[(d)]

* n.d.: not documented

[a] Exact phage titers could be determined.

[b] Exact phage titers could not be determined - cleared lysis zones were visible instead of distinct plaques.

[c] No plaques or lysis zones obtained, titers below limit of detection, i.e. ca. < 10^{2} pfu/ml

[d] EOP values < ca. $3x10^{-8}$
