# Supplementary material for: Characterization of the first Pseudomonas grimontii bacteriophage, PMBT3
Source: Arch Virol. 2021 Aug 4;166(10):2887–94. doi: 10.1007/s00705-021-05173-0 (PMC8421299; doi:10.1007/s00705-021-05173-0)
Supplement: Supplementary file 2 — Supplementary file2 (PDF 125 kb) [file 705_2021_5173_MOESM2_ESM.pdf]

Supplementary Table S2. Overview of the 116 putative ORFs in the genome of phage PMBT3 with their predicted function and the best match result in the databases using BlastP.

ORF	Predicted function (Pfam)	Strand	Position (nt)	Length	conserved domain (acc.)	Best match BlastP [taxon]	E-Value	Identity %
1	phage terminase, large subunit	+	1..1785	594	Terminase_GpA (pfam05876)<br>YbcX (COG5525)	terminase large subunit [*Pseudomonas* phage Lana]	0.0	81.68
2	hypothetical protein	+	1910..3382	490	-	hypothetical protein [*Pseudomonas* phage Lana]	0.0	86.17
3	signal peptide peptidase SppA	+	3394..4890	498	S49_Sppa_36K_type (cd07022)<br>SppA_dom (TIGR00706)	SohB protein [*Pseudomonas* phage Lana]	0.0	74.10
4	hypothetical protein	+	4903..5361	152	-	hypothetical protein [*Pseudomonas* phage Lana]	3e-58	70.39
5	hypothetical protein	+	5401..6579	392	-	hypothetical protein [*Pseudomonas* phage Lana]	0.0	91.54
6	CRISPR-associated<br>protein Cas7	+	6632..7144	170	PTZ00121 (PTZ00121)<br>Cas7_I-E (cd09646)	TolA protein [*Pseudomonas* phage Lana]	5e-33	51.98
7	GTP cyclohydrolase I	+	7155..7640	161	PLN02531 (PLN02531)	hypothetical protein [*Pseudomonas* phage Lana]	3e-79	80.89
8	hypothetical protein	+	7637..8548	303	-	hypothetical protein [*Pseudomonas* phage Lana]	2e-173	77.56
9	hypothetical protein	+	8548..9054	168	-	hypothetical protein [*Pseudomonas* phage Lana]	4e-78	67.07
10	hypothetical protein	+	9051..9503	150	-	hypothetical protein [*Pseudomonas* phage Lana]	4e-84	79.33
11	hypothetical protein	+	9509..10558	349	-	hypothetical protein [*Pseudomonas* phage Lana]	0.0	78.80
12	hypothetical protein	+	10640..11089	149	-	hypothetical protein [*Pseudomonas* phage Lana]	3e-38	48.99
13	hypothetical protein	+	11278..11448	56	-	hypothetical protein [*Pseudomonas stutzeri*]	4e-05	43.10
14	phage tape measure protein	+	11451..16634	1727	tape_meas_TP901 (TIGR01760)<br>PhageMin_Tail (pfam10145)	hypothetical protein [*Pseudomonas* phage Lana]	0.0	46.56
15	phage minor tail protein	+	16638..16985	115	Phage_min_tail (pfam05939)<br>COG4718 (COG4718)	hypothetical protein [*Pseudomonas stutzeri*]	1e-35	48.28
16	phage minor tail protein L	+	17024..17725	233	gp18 (COG4672)<br>phage_tail_L (TIGR01600)	phage minor tail protein L [*Pseudomonas stutzeri*]	1e-111	65.52
17	phage tail assembly protein K	+	17725..18459	244	MPN_NLPC_P60 (cd08073)<br>Rri1 (COG1310)	hypothetical protein [*Pseudomonas stutzeri*]	2e-100	58.92
18	phage tail assembly protein I	+	18456..19049	197	COG4723 (COG4723)	tail assembly protein [*Pseudomonas stutzeri*]	2e-60	49.75

19	phage tail fiber protein	+	19046..22699	1217	COG4733 (COG4733)<br>Phage-tail_3 (pfam13550)	hypothetical protein [*Pseudomonas stutzeri*]	0.0	57.19
20	hypothetical protein	+	22699..23049	116	-	-	-	-
21	hypothetical protein	+	23059..23928	289	-	hypothetical protein [*Pseudomonas stutzeri*]	6e-84	49.13
22	hypothetical protein	+	23942..24760	272	-	hypothetical protein [*Acinetobacter indicus*]	6e-22	49.38
23	peptidoglycan hydrolase YvbX	+	24770..27826	1018	-	hypothetical protein [*Acinetobacter indicus*]	0.0	41.17
24	hypothetical protein	+	27921..28433	170	-	DUF1566 domain-containing protein [*Rugamonas* sp. FT29W]	7e-19	41.32
25	hypothetical protein	+	28528..28926	132	-	hypothetical protein [*Pseudomonas* phage MR14]	6e-35	50.00
26	hypothetical protein	+	28913..29233	106	-	hypothetical protein [*Pseudomonas* phage Lana]	1e-28	81.25
27	lysozyme	+	29223..29765	180	COG3179 (COG3179)<br>chitinase_GH19 (cd00325)	lytic enzyme [*Pseudomonas* phage Lana]	2e-99	78.65
28	DUF2514	+	29765..30298	177	DUF2514 (pfam10721)	hypothetical protein [*Pseudomonas* phage Lana]	5e-49	52.07
29	hypothetical protein	+	30348..30656	102	-	hypothetical protein [*Pseudomonas lini*]	1e-18	46.39
30	hypothetical protein	+	30653..30949	98	-	hypothetical protein [*Pseudomonas fuscovaginae*]	2e-10	53.57
31	hypothetical protein	+	30949..31443	164	-	hypothetical protein [*Pseudomonas otitidis*]	1e-66	63.80
32	hypothetical protein	+	31447..32934	495	-	putative gpH protein [*Pseudomonas* phage SM1]	1e-34	32.34
33	hypothetical protein	+	32931..33584	217	-	DUF1566 protein [*Betaproteobacteria bacterium*]	2e-32	38.76
34	hypothetical protein	-	33647..33925	92	-	hypothetical protein [*Pseudomonas* phage Lana]	6e-09	-38.10
35	hypothetical protein	-	33922..34419	165	-	RNA-binding protein [*Aliifodinibius sediminis*]	7.4	30.48
36	hypothetical protein	-	34406..34672	88	-	hypothetical protein [*Pseudomonas* phage Lana]	3e-10	49.32
37	hypothetical protein	-	34675..34917	80	-	hypothetical protein [*Pseudomonas* phage Lana]	9e-25	63.75
38	DNA repair exonuclease	-	34919..37204	761	SbcC (COG0419)<br>AAA_23 (pfam13476)	plectin 1 isoform 8 [*Pseudomonas* phage Lana]	0.0	57.89
39	hypothetical protein	-	37240..37629	129	-	hypothetical protein [*Kosakonia radicincitans*]	2.9	29.41
40	hypothetical protein	-	37626..38198	190	-	hypothetical protein [*Pseudomonas* phage Lana]	3e-30	51.79

41	DNA repair exonuclease SbcCD nuclease subunit	-	38195..39268	357	SbcD (COG0420)	hypothetical protein [*Pseudomonas* phage Lana]	0.0	76.47
42	DNA ligase, phage-associated	-	39319..40473	384	30 (PHA02587)<br>Adenylation_kDNA_ligase_like (cd07896)	DNA ligase [*Pseudomonas* phage Lana]	0.0	69.79
43	hypothetical protein	-	40507..41028	173	-	hypothetical protein [*Pseudomonas* phage Lana]	2e-93	76.30
44	deoxynucleotide monophosphate kinase	-	41028..41630	200	1 (PHA02575)	hypothetical protein [*Pseudomonas* phage Lana]	7e-81	60.85
45	DNA polymerase III epsilon subunit	-	41627..42373	248	DEDDh (cd06127)<br>PRK07942 (PRK07942)	hypothetical protein [*Pseudomonas* phage Lana]	3e-166	89.52
46	hypothetical protein	-	42370..42753	127	-	hypothetical protein [*Pseudomonas* phage Lana]	1e-11	53.03
47	DNA polymerase I	-	42734..46384	1216	DNA_pol_A (pfam00476)<br>PRK05755 (PRK05755)	DNA polymerase I [*Pseudomonas* phage Lana]	0.0	79.74
48	hypothetical protein	-	46387..46692	101	-	hypothetical protein [*Pseudomonas* phage Lana]	9e-07	43.59
49	thymidylate synthase thyX	-	46704..47393	229	Thy1 (pfam02511)<br>thyX (PRK00847)	thymidylate synthase (FAD) [*Pseudomonas stutzeri*]	1e-93	62.11
50	tellurite resistance protein TerB	-	47451..47903	150	-	tellurite resistance protein [*Pseudomonas* sp. HMWF031]	4e-56	60.40
51	hypothetical protein	-	47960..48226	88	-	hypothetical protein [*Pseudomonas* sp. RW407]	5e-26	58.23
52	nucleotide pyrophosphohydrolase	-	48229..48855	208	-	toxin-antitoxin protein [*Pseudomonas* phage Lana]	2e-71	62.07
53	hypothetical protein	-	48928..49899	323	-	hypothetical protein [*Pseudomonas* phage Lana]	1e-104	55.70
54	SWF/SNF family helicase	-	50026..51741	571	-	putative helicase [*Pseudomonas* phage Lana]	0.0	81.75
55	hypothetical protein	-	51741..51968	75	-	hypothetical protein [*Pseudomonas* phage Lana]	2e-39	82.67
56	RNA polymerase-binding transcription factor	-	51965..54856	963	-	hypothetical protein [*Pseudomonas* phage Lana]	0.0	79.19
57	hypothetical protein	-	54860..55105	81	-	hypothetical protein [*Pseudomonas* phage Lana]	3e-25	62.20
58	DNA helicase RecD	-	55131..56459	442	RecD (COG0507)<br>DEXSc_RecD-like (cd17933)	DNA helicase [*Pseudomonas* phage Lana]	0.0	75.11
59	hypothetical protein	-	56524..56850	108	-	hypothetical protein [*Pseudomonas* phage Lana]	3e-17	41.96
60	hypothetical protein	-	56886..57689	267	-	hypothetical protein [*Pseudomonas* phage Lana]	3e-100	56.18

61	DNA polymerase A	-	57689..58051	120	DNA_pol_A (pfam00476)<br>DNA_pol_A_Aquificae_like (cd08639)	hypothetical protein [*Pseudomonas* phage Lana]	5e-35	57.14
62	hypothetical protein	-	58069..58503	144	-	hypothetical protein [*Pseudomonas* putida]	5e-12	33.09
63	hypothetical protein	-	58500..58832	110	-	hypothetical protein [*Pseudomonas* phage Lana]	3e-41	60.00
64	hypothetical protein	-	58861..59445	194	-	hypothetical protein [*Pseudomonas* phage Lana]	3e-69	54.12
65	hypothetical protein	-	59448..59852	134	-	hypothetical protein [*Pseudomonas* phage Lana]	9e-20	43.69
66	hypothetical protein	-	59861..60319	152	-	hypothetical protein [*Pseudomonas* phage Lana]	1e-24	42.95
67	hypothetical protein	-	60306..60758	150	-	hypothetical protein [*Pseudomonas* phage Lana]	1e-20	37.29
68	hypothetical protein	-	60758..61066	102	-	hypothetical protein [*Pseudomonas synxantha*]	5e-26	50.00
69	hypothetical protein	-	61053..61679	208	-	hypothetical protein [*Pseudomonas* phage Lana]	2e-73	62.12
70	hypothetical protein	-	61676..61960	94	-	hypothetical protein [*Pseudomonas* phage Lana]	1e-18	47.83
71	hypothetical protein	-	61938..62282	114	-	hypothetical protein [*Pseudomonas* phage Lana]	7e-32	53.64
72	chromosome segregation domain protein	-	62270..63460	396	SMC_prok_B (TIGR02168)	hypothetical protein [*Pseudomonas* phage Lana]	3e-46	43.81
73	DNA cytosine methyltransferase	-	63470..65533	687	-	modification methylase [*Pseudomonas* phage Lana]	0.0	78.69
74	hypothetical protein	-	65569..65919	116	-	hypothetical protein [*Pseudomonas* phage Lana]	2e-30	50.00
75	hypothetical protein	-	65993..66166	57	-	hypothetical protein [*Alteromonas* phage JH01]	0.46	54.17
76	hypothetical protein	-	66163..66507	114	-	hypothetical protein [*Pseudomonas* phage Lana]	2e-49	65.77
77	hypothetical protein	-	66500..66649	49	-	DUF5623 protein [unclassified *Bradyrhizobium*]	0.001	48.89
78	hypothetical protein	-	66841..67032	63	-	hypothetical protein [*Pseudomonas* phage Lana]	8e-20	62.90
79	hypothetical protein	-	67022..67315	97	-	hypothetical protein [*Pseudomonas* phage Lana]	6e-30	59.78
80	hypothetical protein	-	67302..67598	98	-	hypothetical protein [*Pseudomonas* phage Lana]	1e-32	55.37
81	hypothetical protein	-	67595..67921	108	-	hypothetical protein [*Pseudomonas* phage Lana]	1e-23	51.06
82	hypothetical protein	-	67932..68126	64	-	-	-	-
83	hypothetical protein	-	68284..68475	63	C2B_Rabphilin_Doc2 (cd08384)	hypothetical protein [*Pseudomonas* phage Lana]	2e-04	41.43

84	hypothetical protein	-	68472..69143	223	-	hypothetical protein [*Pseudomonas* phage Lana]	2e-108	66.07
85	hypothetical protein	-	69211..69381	56	-	DUF4349 domain-containing protein [*Janibacter massiliensis*]	0.17	42.50
86	hypothetical protein	-	69381..69749	122	-	hypothetical protein [*Pseudomonas* phage Lana]	4e-29	49.58
87	hypothetical protein	-	69749..69976	75	-	hypothetical protein [*Caulobacteraceae bacterium*]	1e-05	31.94
88	hypothetical protein	-	69958..70203	81	-	hypothetical protein [*Geminicoccus* sp. CPCC 101082]	3e-16	43.06
89	hypothetical protein	-	70181..70486	101	-	hypothetical protein [*Pseudomonas* phage Lana]	4e-11	37.93
90	hypothetical protein	-	70486..70827	113	-	MULTISPECIES: hypothetical protein [*Pseudomonas*]	3e-08	52.54
91	hypothetical protein	-	70827..70946	39	-	-	-	-
92	hypothetical protein	-	70936..71166	76	-	endosomal targeting BRO1-like protein [*Theobroma cacao*]	0.34	41.38
93	hypothetical protein	-	71168..71419	83	-	-	-	-
94	hypothetical protein	-	71416..72381	321	-	hypothetical protein CTY35_03470 [*Methylotenera* sp.]	1e-20	28.66
95	hypothetical protein	-	72378..72530	50	-	-	-	-
96	DNA binding regulator	-	72714..73121	135	LytTR (pfam04397; smart00850)	hypothetical protein [*Pseudomonas* sp. QS1027]	3e-08	35.34
97	hypothetical protein	-	73108..73635	175	-	hypothetical protein Zuri_31 [*Pseudomonas* phage Zuri]	4e-08	32.04
98	hypothetical protein	-	73598..74110	170	-	hypothetical protein [*Pseudomonas* phage Lana]	7e-32	58.82
99	hypothetical protein	-	74110..74649	179	-	hypothetical protein [*Pseudomonas* phage Lana]	3e-29	65.43
100	hypothetical protein	-	74646..74963	105	-	hypothetical protein [*Pseudomonas* phage Lana]	7e-30	51.43
101	hypothetical protein	-	74953..75261	102	-	-	-	-
102	hypothetical protein	-	75263..75658	131	-	hypothetical protein [*Pseudomonas sagittaria*]	8e-04	44
103	hypothetical protein	-	75651..76910	419	-	hypothetical protein [*Pseudomonas japonica*]	1e-09	48.48
104	hypothetical protein	-	76916..77446	176	-	-	-	-
105	hypothetical protein	-	77456..78712	418	-	hypothetical protein [*Pseudomonas* phage Lana]	8e-83	40.10
106	hypothetical protein	+	79526..79771	81	-	-	-	-
107	hypothetical protein	-	79931..80311	126	-	hypothetical protein [*Pseudomonas* phage Lana]	1e-28	42.75

108	phage portal protein	-	80315..80788	157	COG5511 (COG5511)	hypothetical protein [*Pseudomonas* phage Lana]	9e-52	59.87
109	hypothetical protein	-	80983..81273	96	-	hypothetical protein [*Pseudomonas* phage Lana]	2e-27	56.82
110	hypothetical protein	-	81329..82096	255	-	hypothetical protein [*Pseudomonas* phage Lana]	3e-92	57.45
111	hypothetical protein	+	83000..83848	282	-	hypothetical protein [*Pseudomonas* phage Lana]	2e-34	75.95
112	hypothetical protein	+	83980..84138	52	-	-	-	-
113	hypothetical protein	+	84131..85060	309	-	-	-	-
	tRNA—Pro	+	85132..85205					
	tRNA-Gln	+	85214..85289					
	tRNA-Met	+	85417..85489					
114	integration host factor subunit beta	+	85843..86133	96	IHF (cd13832)	hypothetical protein [*Pseudomonas* phage Lana] Bac_DNA_binding (pfam00216)	1e-45	73.96
115	hypothetical protein	+	86133..86729	198	-	hypothetical protein [*Pseudomonas* phage Lana]	2e-68	62.36
116	hypothetical protein	+	86726..87190	154	-	hypothetical protein [*Pseudomonas* phage Lana]	0.001	37.98
