# Supplementary material for: Characterization of the first Pseudomonas grimontii bacteriophage, PMBT3
Source: Arch Virol. 2021 Aug 4;166(10):2887–94. doi: 10.1007/s00705-021-05173-0 (PMC8421299; doi:10.1007/s00705-021-05173-0)
Supplement: Supplementary file 3 — Supplementary file3 (PDF 111 kb) [file 705_2021_5173_MOESM3_ESM.pdf]

Supplementary Table S3. Predicted host-dependent promoter sequences in the genome of phage PMBT3 discovered by Kodon.

Promoter	Strand	Position (nt)	Promoter sequence
1	-	50000..50029	TTGACAGAGGGCAAGGCTGCTCTGTATAAA
2	-	78842..78868	TAGACAGAAACAGGGCGAAAATATAGT
3	+	83823..83851	TTGACATTACGAAAGTGCCGAGCTAAAGT

Supplementary Table S4. Predicted rho-independent terminators (ΔG less than -10 kcal mol^{-1}), where loops are in red and stems in blue in the genome of phage PMBT3 using ARNold.

Terminator	Strand	Position (nt)	Terminator sequence	ΔG (kcal mol^{-1})
1	+	6594..6617	GGGCCGCTTCGGTGGCCCTTATTT	-14.5
2	+	33619..33642	GGGGCAGCGATCGCTGCCCCTTTT	-17.8
3	-	47412..47436	GCTCCCGCCTTCTGGCGGGAGCTTT	-17.3
4	-	48880..48910	GGCCCGACAGGTAACACTGTCGGGCTTTTTT	-15.1
5	-	80922..80954	GCCAGGCACGCCCTAACGTGCCTGGCcTTTTTT	-14.3
6	+	83889..83925	CCCAAGGGCCTAGTCCTCCCTTGGGgTCTTTTTTATT	-13.9
7	+	85673..85707	CCCCATGCGCGGCGCGCATGGGGTTTCTTTTATTT	-15.2
