# Supplementary material for: Characterization of the first Pseudomonas grimontii bacteriophage, PMBT3
Source: Arch Virol. 2021 Aug 4;166(10):2887–94. doi: 10.1007/s00705-021-05173-0 (PMC8421299; doi:10.1007/s00705-021-05173-0)
Supplement: Supplementary file 4 — Supplementary file4 (PDF 30 kb) [file 705_2021_5173_MOESM4_ESM.pdf]

Supplementary Table S5. GenBank protein accession numbers **(**GBANs) of bacterial and phage terminase large subunit (TerL)-like proteins used for alignment.

**Microorganism**	**Strain**	**GBAN**
Pseudomonas phage	Lana	YP_009820332.1
Pseudomonas balearica	n.a.	MBC7198117.1
Chloroflexi bacterium	n.a.	RMG71620.1
Vibrio phage	SHOU24	YP_009006476.1
Phycisphaerales bacterium	n.a.	HEC04503.1
Desulfobulbaceae bacterium	n.a.	HHL35205.1
Actinobacteria bacterium	n.a.	RLE20876.1
Methylovulum miyakonense	n.a.	WP_019865150.1
Gammaproteobacteria bacterium	n.a.	NLY59328.1

Supplementary Table S6. GBANs of bacterial and phage TerB-like proteins used for alignment.

**Microorganism**	**Strain**	**GBAN**
Pseudomonas jessenii	n.a.	WP_090455588.1
Pseudomonas sp.	GM55	WP_008015874.1
Enterobacteria phage	P7	YP_009914545.1
Pseudomonas putida	n.a.	WP_046786640.1
Pseudomonas fluorescens	n.a.	WP_151213795.1
Pseudomonas sp.	HMWF031	PTU03211.1
Enterobacter cloaceae	n.a.	WP_063928746.1
Escherichia coli	MS 57-2	EGB75990.1
Pseudomonas phage	Lana	YP_009820378.1

Supplementary Table S7. GBANs and characteristics of *Pseudomonas* phage genomes used for phylogenetic analysis.

***Pseudomonas* Phage**	**Family/ Genus**	**Bacterial host**	**Genome size (bp)**	**GBAN**
Lana	*Siphoviridae*	*Pseudomonas* sp.	88403	MK473373.2
PMBT14	*Siphoviridae*	*Pseudomonas fluorescens* ATCC 13525	47820	MG596800.2
PEV2	*Podoviridae*/ *Litunavirus*	*Pseudomonas aeruginosa* Kutter	72697	KU948710.1
phi-2	*Podoviridae*/ *Phikmvvirus*	*Pseudomonas fluorescens* SBW25	43144	FN594518.1
Pf-10	*Podoviridae*/ *Teseptimavirus*	*Pseudomonas fluorescens* BIM B-582	39167	NC_027292.1
UFV-P2	*Podoviridae*/ *Vicosavirus*	*Pseudomonas fluorescens*	45517	NC_018850.2
phiIBB-PF7A	*Podoviridae*/ *Teseptimavirus*	*Pseudomonas fluorescens*	40973	NC_015264.1
DVM-2008	*Myoviridae*	*Pseudomonas fluorescens* Q8r1-96	22689	EU982300.1
Phi-S1	*Podoviridae*/ *Teseptimavirus*	*Pseudomonas fluorescens*	40192	NC_021062.1
VW-6S	*Siphoviridae*	*Pseudomonas fluorescens* W-6	37917	MF975720.1
UNO-SLW1	*Podoviridae*/ *Teseptimavirus*	*Pseudomonas* sp.	39215	KX431888.1
UNO-SLW2	*Podoviridae*/ *Teseptimavirus*	*Pseudomonas* sp.	39167	KX449361.1
UNO-SLW3	*Podoviridae*/ *Teseptimavirus*	*Pseudomonas* sp.	39092	KX449362.1
UNO-SLW4	*Podoviridae*/ *Teseptimavirus*	*Pseudomonas* sp.	39136	KX449363.1
BIM BV-46	*Podoviridae*/ *Teseptimavirus*	*Pseudomonas fluorescens* BIM B-582	38860	MT094431.1
Noxifer	*Myoviridae*/ *Noxifervirus*	*Pseudomonas fluorescens* SBW25	278136	NC_041994.1

67PfluR64PP	*Podoviridae*/ *Teseptimavirus*	*Pseudomonas fluorescens*	40748	MH179478.2
22PfluR64PP	*Podoviridae*/ *Teseptimavirus*	*Pseudomonas fluorescens*	40822	MH179472.2
98PfluR60PP	*Podoviridae*	*Pseudomonas fluorescens*	74361	MH179480.1
Phabio	*Myoviridae*/ *Phikzvirus*	*Pseudomonas fluorescens* SBW25	309157	MF042360.1
PFP1	*Podoviridae*/ *Teseptimavirus*	*Pseudomonas fluorescens*	40914	MH268168.1
PPSC2	*Myoviridae*/ *Otagovirus*	*Pseudomonas fluorescens* SA1	97330	MF893340.1
VW-6B	*Siphoviridae*	*Pseudomonas fluorescens* W-6	35306	MF975721.1
SCYZ1	*Podoviridae*/ *Krylovvirus*	*Pseudomonas fluorescens*	47475	MH518298.3
phCDa	*Podoviridae*	*Pseudomonas fluorescens* CS1	72821	MH382836.1
OBP	*Myoviridae*	*Pseudomonas fluorescens* Pf1.1	284757	NC_016571.1
VSW-3	*Podoviridae*/ *Napahaivirus*	*Pseudcmonas fluorescens* SW-3	40556	NC_041885.1
BIM BV-45	*Podoviridae*/ *Bifseptvirus*	*Pseudomonas fluorescens* BIM B-582	40565	MT094430.1
Skulduggery	*Podoviridae*	*Pseudomonas fluorescens* SBW25	62978	MF042361.1
KNP	*Podoviridae*/ *Teseptimavirus*	*Pseudomonas fluorescens* group	40491	KY798121.1
WRT	*Podoviridae*/ *Teseptimavirus*	*Pseudomonas fluorescens* group	40214	KY798120.1
Pf1 ERZ-2017	*Podoviridae*/ *Teseptimavirus*	*Pseudomonas fluorescens*	39195	MG250485.1
