# Supplementary material for: Characterization of the first Pseudomonas grimontii bacteriophage, PMBT3
Source: Arch Virol. 2021 Aug 4;166(10):2887–94. doi: 10.1007/s00705-021-05173-0 (PMC8421299; doi:10.1007/s00705-021-05173-0)
Supplement: Supplementary file 5 — Supplementary file5 (PDF 725 kb) [file 705_2021_5173_MOESM5_ESM.pdf]

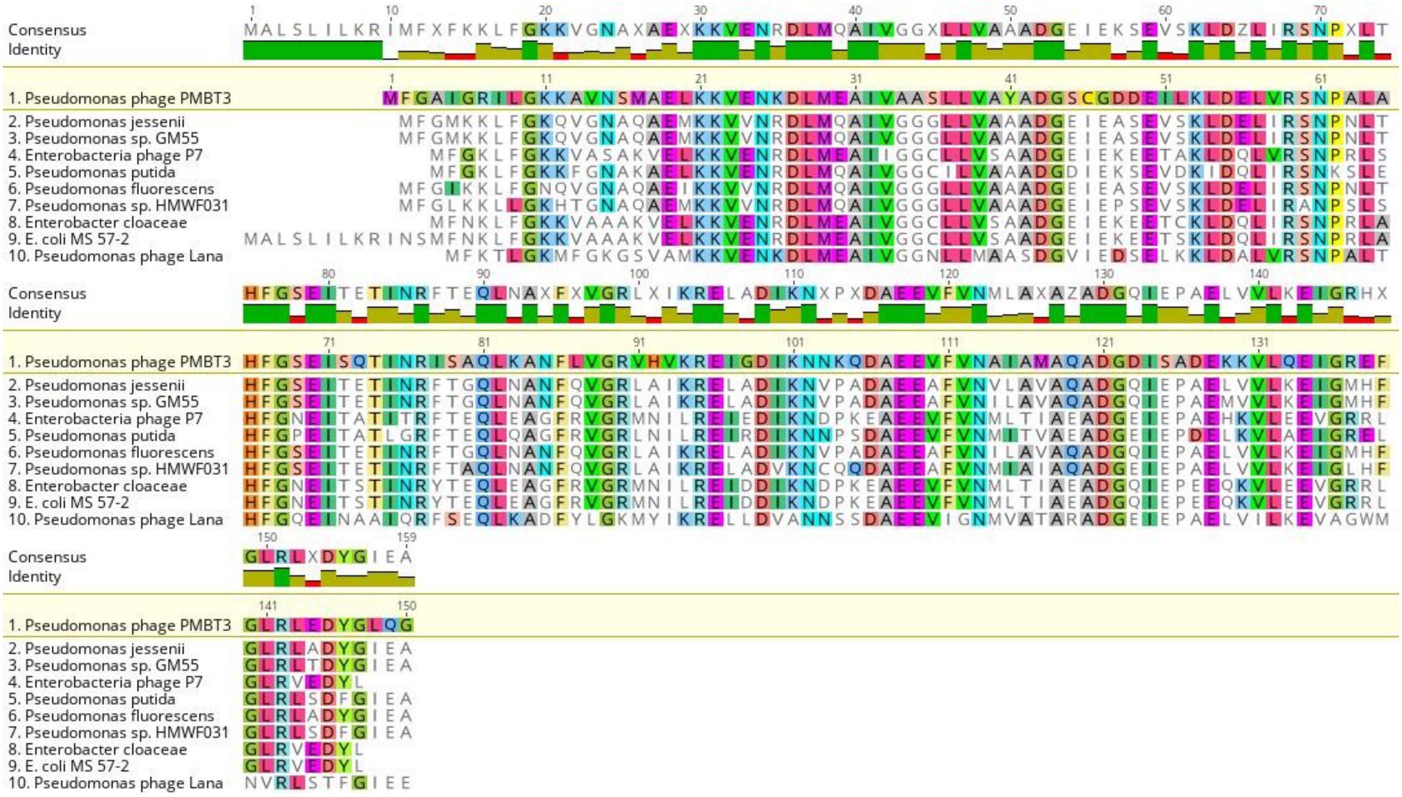

Fig. S1. Amino acid sequence alignment of putative TerB from phage PMBT3 with the TerB proteins of *Pseudomonas* phage Lana, different bacteria (various *Pseudomonas* species, *Enterobacter cloacae* and *Escherichia coli*) and one Enterobacteria phage. These proteins represent closely related proteins showing e-values from 1e-53 to 3e-43. Coloured amino acids indicate a sequence consensus; colourless amino acids indicate differences between the sequences. The consensus sequence is shown on top of the alignment. The alignment was performed with Geneious 11.0.2.

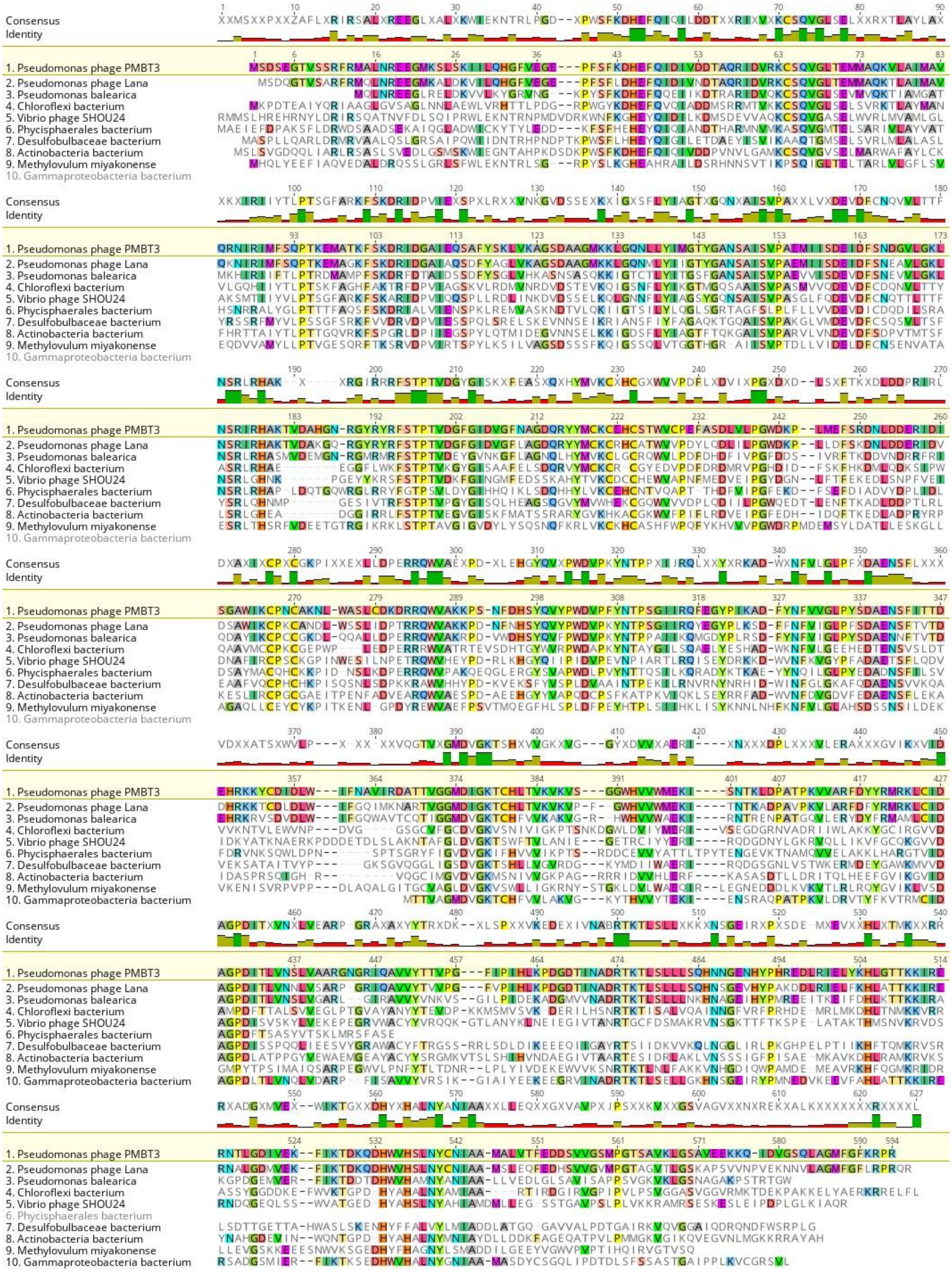

Fig. S2. Amino acid sequence alignment of putative terminase large subunit (TerL) from phage PMBT3 with deduced proteins of different bacteria and phages. These proteins represent the most closely related proteins based on the e-value. Coloured amino acids indicate a sequence consensus; colourless amino acids indicate differences between the sequences. The consensus sequence is shown on top of the alignment. The alignment was performed with Geneious 11.0.2.
